# Isoxanthohumol improves hepatic lipid metabolism via regulating the AMPK/PPARα and PI3K/AKT signaling pathways in hyperlipidemic mice

**DOI:** 10.1002/fsn3.4449

**Published:** 2024-09-10

**Authors:** Yu Gao, Qilong Zhou, Huiqing Wang, Guang Xin, Tao Wang, Kun Zhang, Xiuxian Yu, Ao Wen, Qiuling Wu, Xiaojuan Li, Yijiang Liu, Wen Huang

**Affiliations:** ^1^ West China Center of Excellence for Pancreatitis, Institute of Integrated Traditional Chinese and Western Medicine, Natural and Biomimetic Medicine Research Center, Tissue‐Orientated Property of Chinese Medicine Key Laboratory of Sichuan Province, West China School of Medicine West China Hospital, Sichuan University Chengdu People's Republic of China; ^2^ Department of Pediatrics West China Second University Hospital, Sichuan University Chengdu People's Republic of China

**Keywords:** hyperlipidemia, isoxanthohumol, molecular docking, network pharmacology

## Abstract

Hyperlipidemia presents a significant global healthcare challenge, necessitating innovative therapeutic strategies for more effective outcomes. Recent studies have highlighted the beneficial impact of moderate beer intake on metabolic diseases. The purpose of this research is to explore the possible molecular mechanisms of isoxanthohumol (IXN), the major hop flavonoid in beer, in the treatment of hyperlipidemia. The mice model of acute hyperlipidemia was constructed by intraperitoneal injection of Triton WR‐1339. The therapeutic effect of IXN was assessed by biochemical and histological analyses. Furthermore, comprehensive data mining across various public databases was conducted to identify underlying therapeutic targets of IXN on hyperlipidemia. A protein–protein interaction network was constructed to pinpoint hub targets, and subsequent GO and KEGG enrichment analyses were used to elucidate underlying biological functions. Molecular docking was utilized to validate the binding affinity between hub targets and IXN. Western blotting analysis further verified the protein expression of potential IXN targets. IXN administration significantly improved blood lipid and hepatic lipid levels, alongside increased SOD activity and decreased MDA content in hyperlipidemia mice. Histological analyses, including H&E and Oil Red O staining, showed the improvement of hepatic steatosis with IXN treatment. At the molecular level, IXN significantly increased protein levels of p‐AMPK, PPARα, p‐PI3K, and p‐AKT. IXN activates AMPK/PPARα and PI3K/AKT signaling pathways, leading to reduction in lipid accumulation and oxidative stress, and ultimately ameliorating hyperlipidemia.

## Graphical abstract

Isoxanthohumol significantly improves blood lipid levels, attenuates hepatic steatosis, and mitigates oxidative stress in hyperlipidemic mice. These beneficial effects are possibly attributed to the upregulation of the AMPK/PPARα and PI3K/AKT signaling pathways.

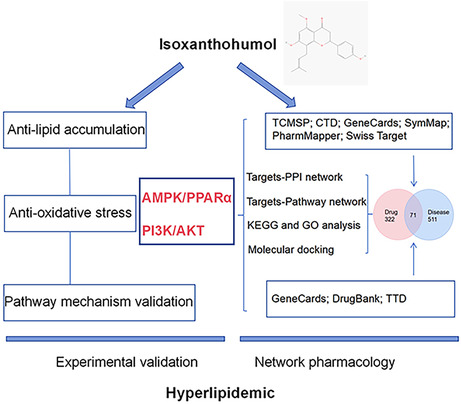

## 1 INTRODUCTION

Hyperlipidemia, a widespread health concern in contemporary society, is exacerbated by unhealthy lifestyle choices marked by poor dietary habits and insufficient physical activity. Its diagnosis primarily hinges on identifying abnormal deviations in serum lipids, encompassing elevated levels of triacylglycerols (TG), total cholesterol (TC), and low‐density lipoprotein cholesterol, as well as reduced high‐density lipoprotein cholesterol. The implications of hyperlipidemia and its associated lipid disorders are profound, contributing significantly to serious health issues such as atherosclerosis, stroke, myocardial infarction, pancreatitis, diabetes, and kidney failure (Bozkurt et al., 2016; D'Elia et al., 2022; Libby et al., 2019; Sandhu et al., 2011). Across diverse cultures, a direct linear correlation between cholesterol levels and coronary heart disease mortality has been established (Verschuren et al., 1995), emphasizing the pivotal role of normalizing serum lipid levels in averting chronic diseases, particularly cardiovascular diseases. In the landscape of lipid‐lowering therapies, statins and fibrates stand out as extensively employed interventions globally. However, their widespread use is hindered by side effects and suboptimal tolerance in certain patient populations (Liu et al., 2019; Okopień et al., 2018).The imperative to explore alternative approaches persists in addressing the multifaceted challenges posed by hyperlipidemia and its associated health risks.

Moreover, moderate beer consumption, defined as women drinking one glass per day and men drinking one to two cups per day, has emerged as a noteworthy factor associated with lower risk of cardiovascular morbidity and various metabolic health benefits (Arranz et al., 2012; Marcos et al., 2021). Beer serves as a primary dietary source of Isoxanthohumol (IXN), a compound obtained from hop during the brewing process (Martinez‐Gomez et al., 2020; Quifer‐Rada et al., 2015). IXN, as well as a common active ingredient in many Chinese herbal medicines (Lin et al., 2022), exhibits diverse biological activities, including anti‐tumor properties, antioxidation, inhibition of insulin resistance, suppression of preadipocyte differentiation, and induction of apoptosis in mature adipocytes (Krajnović et al., 2016; Negrão et al., 2013; Yamashita et al., 2020; Yang et al., 2007). Furthermore, recent studies highlight the potential regulatory role of IXN in chronic obesity model (Watanabe et al., 2023). Despite this knowledge, the specific beneficial effects of IXN on abnormalities associated with hyperlipidemia remain unclear.

In the realm of experimental animal models, Triton WR‐1339 (triton), a commonly used nonionic detergent, was believed to have the ability to induce acute hyperlipidemia by blocking the clearance of TG‐rich lipoproteins and increasing hepatic cholesterol biosynthesis (Filho et al., 2017; Goldfarb, 1978; Zhao et al., 2014). Considering the liver's crucial role in cholesterol transport, metabolism, and excretion, our study aims to explore hepatic lipid metabolism in the context of acute hyperlipidemia (Zarzecki et al., 2014). Specifically, we focus on evaluating the impact of IXN on metabolic disorders and tissue impairment induced by acute hyperlipidemia. Furthermore, we employ network pharmacology and molecular docking to unravel the underlying mechanisms of observed efficacies by IXN. This comprehensive approach seeks to enhance our understanding of the interplay between IXN and acute hyperlipidemia, offering valuable insights into therapeutic possibilities for metabolic disorders associated with elevated lipid levels.

## 2 MATERIALS AND METHODS

### 2.1 Drugs and reagents

Triton WR1339 (HY‐B1068) was sourced from MCE (Shanghai, China), while IXN (B21530) was purchased from Shanghai Ye Yuan Co., Ltd. (Shanghai, China), and Fenofibrate from Shanghai Hengshan Pharmaceutical Industry (Shanghai, China).

### 2.2 Network pharmacology analysis and molecular docking of IXN

#### 2.2.1 Structural information

We utilized the PubChem database (https://pubchem.ncbi.nlm.nih.gov/) for accessing information containing biological attributes and chemical compositions of small molecules, including IXN (Wang et al., 2009).

#### 2.2.2 IXN‐associated targets' prediction

To acquire potential targets of IXN, the databases for TCMSP, CTD (https://ctdbase.org), GeneCards (https://www.genecards.org/), SymMap (http://www.symmap.org/), PharmMapper (http://www.lilab‐ecust.cn/pharmmapper/), and Swiss Target Prediction (http://swisstargetprediction.ch/) were used (Daina et al., 2019; Davis et al., 2021; Liu et al., 2010; Ru et al., 2014; Safran et al., 2010; Wu et al., 2019). The drug name “Isoxanthohumol (IXN)” was entered into the search window in the TCMSP, CTD, GeneCards, and SymMap databases. The mol2 format file and SMILES structural formula were input into PharmMapper and Swiss Target Prediction for target prediction. For each database, species were restricted to “Homo sapiens.” All targets were converted into gene names by Uniport (https://www.uniprot.org/). Finally, the retrieved targets were combined and deduplicated.

#### 2.2.3 Hyperlipidemia‐associated targets’ prediction

The hyperlipidemia‐associated targets were obtained from the GeneCards, DrugBank (https://www.drugbank.ca/), and TTD (http://db.idrblab.net/ttd/) databases (Li et al., 2018; Wishart et al., 2018). The search terms were “hyperlipidemia,” “hyperlipidemias,” and “Dyslipidemia” according to the characteristics of different databases, and only Homo sapiens proteins were selected. We then selected potential disease genes based on database scoring criteria. Additionally, we obtained detailed gene annotations from UniProt to ensure a comprehensive analysis.

#### 2.2.4 Construction of protein–protein interaction (PPI) network

The drug‐disease common targets were further analyzed using the String database (https://cn.string‐db.org/) to forecast protein–protein interactions (PPIs) in H. sapiens, focusing on those with a binding score of 0.9 or higher. Then, PPI networks were visualized by Cytoscape 3.9.1 (Shannon et al., 2003). Key genes were predicted by utilizing Cytoscape plug‐in CytoNCA, and betweenness centrality (BC) of the hub genes was calculated. BC, a critical topological metric, allowed us to pinpoint and rank the network's most interconnected nodes or hubs, providing a hierarchy of candidate reference genes based on their BC values (You et al., 2010).

#### 2.2.5 Functional enrichment analyses

Kyoto Encyclopedia of Genes and Genomes (KEGG) pathway enrichment analysis was carried out utilizing DAVID database (https://david.ncifcrf.gov) (Dennis Jr. et al., 2003). The former 20 enriched pathways were selected to create a pathway‐target network to evaluate the hub targets using Cytoscape software. Also, the DAVID database was employed for gene ontology (GO) enrichment analysis, covering aspects such as biological processes (BP), cellular components (CC), and molecular functions (MF) (Gene Ontology Consortium., 2006). *p*‐value less than 0.05 were deemed significant. The enrichment results were validated using Metascape database (https://metascape.org) (Zhou et al., 2019). For visualization, we utilized the Omicshare platform (https://www.omicshare.com/), which effectively represents the findings in bubble charts.

#### 2.2.6 Molecular docking

Molecular docking could simulate the interaction of protein targets and small‐molecule compounds (Saikia & Bordoloi, 2019). Sybyl 2.0 software was used to determine the prospective binding efficiency between hyperlipidemia targets and IXN. Docking outcomes were quantitatively assessed, the score >4.52 represents a certain binding activity, and >5.0 represents a good binding activity (a higher score indicates stronger binding activity).

### 2.3 Animal experiments

#### 2.3.1 Grouping and treatment

C57BL/6 mice (male, 8 weeks old), provided by Chengdu Dashuo Experimental Animal Co., Ltd. (Chengdu, China), underwent a 7‐day acclimation before experimental commencement. Mice were fed under regulated conditions, including the temperature of 23 ± 2°C, humidity of 50 ± 10%, and light–dark cycle (12 h light/12 h dark). The mice were randomly allocated into five groups, each consisting of seven mice: the normal control (NC) group, model (Triton) group, positive control (PC) group (Fenofibrate, 50 mg/kg), low‐dose IXN (L‐IXN) group (IXN, 10 mg/kg), and high‐dose IXN (H‐IXN) group (IXN, 20 mg/kg). First, the NC and Triton groups were treated with equal volume of solvents (PBS) by oral gavage, and PC and IXN treatment groups were treated with the indicated medicine by oral gavage at the indicated time points (12 h before, 2 and 22 h post‐Triton WR‐1339 administration). Then, a single intraperitoneal injection of Triton WR‐1339 (350 mg/kg body weight) was given to the Triton, PC, and IXN treatment groups to establish the hyperlipidemia models. The NC group received an equivalent volume of intraperitoneal PBS injection at the corresponding time point. Two hours after the last treatment, mice were sacrificed, and tissue samples were extracted for further analysis. Serum samples were collected after fasting for at least 4 h. All animal experiments were conducted in compliance with the guidelines and regulations of China regarding the use and welfare of experimental animals (GB/T 35892–2018 and GB/T 35823–2018). The experimental protocols received approval from the Ethics Committee of the West China Hospital of Sichuan University (Ethics record number: 20220214019).

#### 2.3.2 Biochemical analysis

Twenty‐four hours following Triton WR‐1339 administration, we performed cardiac punctures on anesthetized mice to procure blood samples, which were then centrifuged (4000 rpm for 10 min) to obtain serum. The levels of TG, TC, aspartate transaminase (AST), and alanine aminotransferase (ALT) in the serum were evaluated using an automatic biochemical analyzer (Cobas702, Roche, Mannheim, Germany), following the manufacturer's protocol. Serum contents of malondialdehyde (MDA) and superoxide dismutase (SOD) were assessed via detection kits (Jian‐Cheng, Nan Jing, China).

TG and TC were extracted from mouse liver and quantitated by use of TG (Mlbio, Shanghai, China) and TC (Elabscience, Wuhan, China) assay kits following manufacturer's instructions.

#### 2.3.3 Histological analysis

Hematoxylin–Eosin (H&E) was employed to check the histopathological alterations of the liver. Lipid accumulation was visualized using Oil Red O (ORO) staining. H&E: liver tissues were processed for fixation (4% formaldehyde), dehydration, paraffin embedding, and sectioning. The slices were dewaxed with xylene, rehydrated with graded ethanol, and then stained with hematoxylin and eosin in turn. ORO: frozen sections were fixed with paraformaldehyde, rinsed with 60% isopropanol, stained with newly prepared ORO working solution for 10 min, and rinsed with 60% isopropanol. We examined all pathological slides under a light microscope (AX10 imager A2/AX10 cam HRC; Carl Zeiss, Germany), selecting three fields of view per section, and magnified them to 200× and 400× to assess the extent of lipid deposits. The histopathological scoring of liver was quantitatively assessed according to the nonalcoholic fatty liver disease activity score (NAS) (Kleiner et al., 2005), encompassing steatosis levels, lobular inflammation, and cellular ballooning (Table S1). The lipid area was quantified using ImageJ software (Schneider et al., 2012).

#### 2.3.4 Western blot

First, frozen liver tissues were pulverized and sonicated in RIPA buffer (Beyotime, China). The homogenate was put on ice for 10 min and then centrifuged at 12000 rpm at 4°C for 10 min. Supernatant was kept at −80°C. Protein concentration was measured using the BCA assay (Beyotime, China), and the lysate was boiled in fivefold loading buffer (Beyotime, China) for 5 min. Then, the denatured protein lysates were separated by 10–15% (the concentration of the gel was decided according to the protein molecular weight) SDS‐PAGE gel (Epizyme, Shanghai), followed by transferring to PVDF membranes (Millipore, America). Membranes were blocked with 5% milk in TBST for 1 h at room temperature. After that, the membranes were incubated with AMPK (1:1000; CST, 2532), p‐AMPK (1:1000; CST, 2535), AKT (1:1000; CST, 9272), p‐AKT (1:1000; CST, 9271), PI3K (1:1000; CST, 4257), p‐PI3K (1:1000; CST, 4228), PPARα (1:1000; Abclonal, A18252), and β‐actin (1:5000; Abclonal, AC026) antibodies at 4°C overnight. Subsequently, membranes were incubated with either goat anti‐mouse IgG or goat anti‐rabbit IgG (Beyotime, China) at room temperature for 1 h. The developed blots were visualized with an ECL chemiluminescent kit (Millipore, America), and protein expression was normalized to β‐actin, quantified by Image J software.

### 2.4 Statistical analysis

Statistical analysis was performed using GraphPad Prism version 8 (San Diego, CA, USA). The significance of our data across multiple samples was determined through one‐way analysis of variance (ANOVA), with *p*‐value less than 0.05 deemed indicative of statistical significance.

## 3 RESULTS

### 3.1 Therapeutic effects and hepatotoxicity evaluation of IXN in triton‐induced hyperlipidemia mice

**FIGURE 1:**
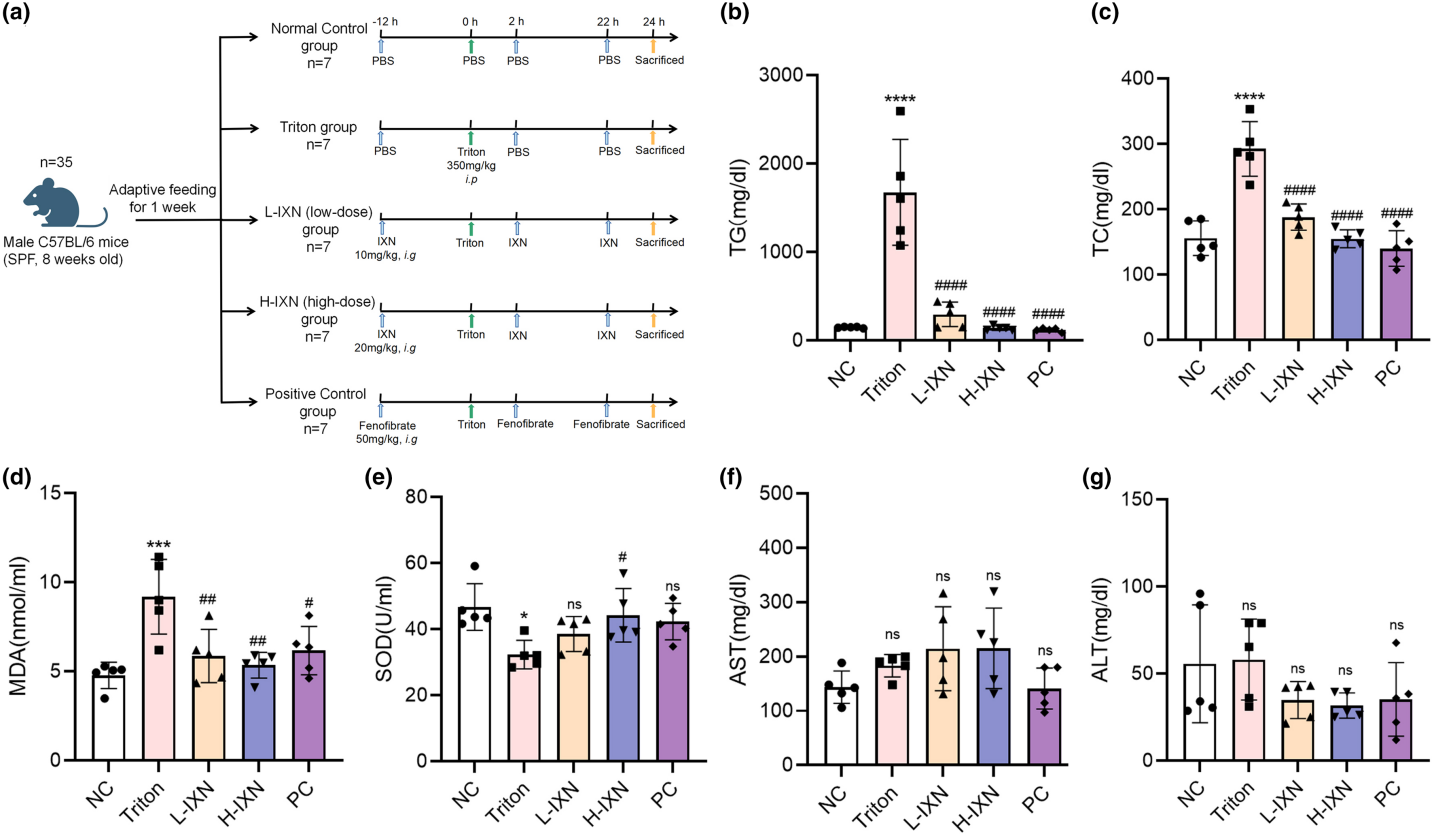
Flowchart of the animal experiments and effects of IXN on serum TG, TC, MDA, SOD, AST, and ALT. (a) Flowchart of the animal experiments. (b, c) Serum lipid metabolism indicators (TG and TC). (d, e) Serum oxidative stress indicators (MDA and SOD). (f, g) Serum liver injury indicators (AST and ALT). Data are shown as mean ± SD for *n* = 5. *p < .05, ***p < .001, ****p < .0001 versus NC group; ^#^ *p* < .05, ^##^ *p* < .01, ^####^ *p* < .0001 versus Triton group; NS: Denotes no significant difference. ALT, alanine aminotransferase aspartate; AST, aspartate transaminase;IXN, isoxanthohumol; MDA, malondialdehyde; SOD, superoxide dismutase; TC, total cholesterol; TG, triacylglycerols.

We induced hyperlipidemia in mice using Triton WR‐1339. Scheme for animal experiments is shown in Figure 1a. At present, serum TG and TC are often used as test indicators of hyperlipidemia. Investigation of blood lipid levels revealed a substantial increase of approximately tenfold in TG and twofold in TC in the Triton group, confirming the successful establishment of the hyperlipidemia model (Figure 1b,c). Treatment with both low and high doses of IXN led to obvious inhibition of TC and TG levels compared to the Triton group, implying the effective reversal of abnormalities in serum TC and TG levels. Notably, no differences were found in terms of TG or TC between IXN and the PC groups, underscoring the effectiveness of IXN. Furthermore, the higher dose of IXN exhibited superior effects compared to the lower dose, highlighting dose‐dependent efficacy (Figure 1b,c).

In the context of oxidative stress, a recognized early event in the hyperlipidemia development (Boudina et al., 2012; Yang et al., 2008), MDA, and SOD levels were assessed. Compared with the NC group, the Triton group displayed a significant increase in MDA levels and a decrease in SOD levels. Treatment with IXN resulted in a decrease in MDA content across all groups, while SOD activity significantly increased, particularly in the H‐IXN group, surpassing the PC group. These serological results collectively support the efficacy of IXN in mitigating oxidative stress, with higher doses (20 mg/kg) exhibiting superior effects compared to fenofibrate (Figure 1d,e). Importantly, the assessment of hepatotoxicity through analysis of AST and ALT levels in plasma revealed that no significant differences among these groups (Figure 1f,g). Additionally, we compared AST and ALT levels in normal and high‐dose IXN alone administration mice without hyperlipidemia (Figure S1). Our results demonstrated that IXN intervention did not cause liver damage.

### 3.2 IXN ameliorates hepatic lipid metabolism disorder induced by triton in mice

**FIGURE 2:**
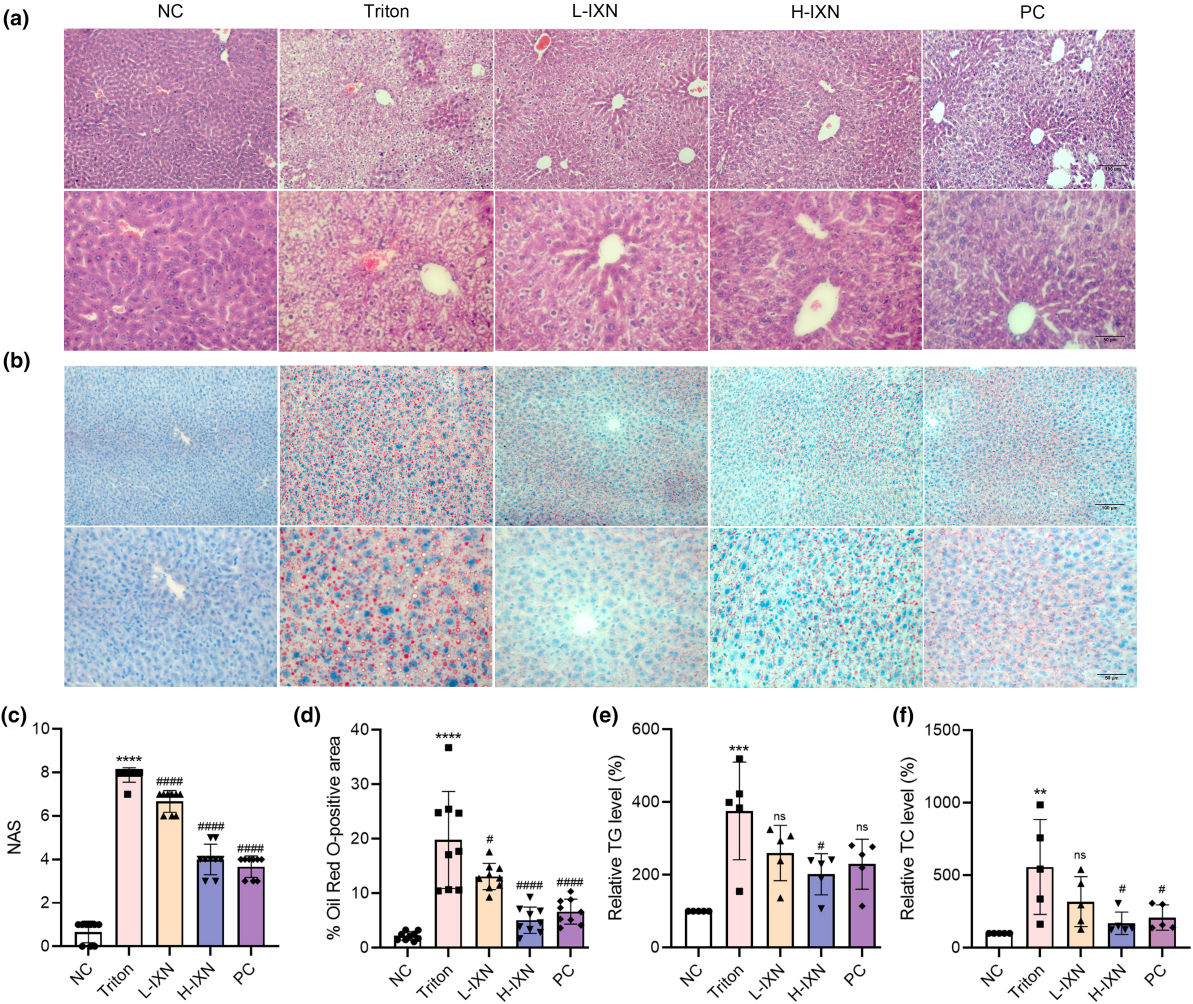
Effects of IXN on histological changes in liver tissues. (a) Representative photomicrographs of liver histology (H&E) from each group. Scale bar, 50 and 100 μm (insets). (b) Oil Red O staining of lipid droplets liver. Scale bar, 50 and 100 μm (insets). (c) NAS (*n* = 9). (d) Quantification of relative area positive for Oil Red O staining (*n* = 9). (e) Hepatic total cholesterol (*n* = 5). (f) Hepatic triglyceride content (*n* = 5). All data represent mean ± SEM. **p < .01, ***p < .001, ****p < .0001 versus NC group; ^#^ *p* < .05, ^####^ *p* < .0001 versus triton group; NS: Denotes no significant difference. IXN, isoxanthohumol; NAS, nonalcoholic fatty liver disease activity score.

Hepatic steatosis and lipid deposition were assessed by H&E and ORO. In the NC group, hepatocytes exhibited an orderly arrangement, while the Triton group displayed significant lipid deposition and vacuolar degeneration around the central vein. IXN treatment effectively restored liver morphology to that of the NC, as evidenced by lower NAS scores, indicating fewer signs of ballooning, inflammation, or steatosis in IXN groups compared to the Triton group (Figure 2a,c).

ORO staining further supported these findings, as the hepatocytes of the Triton group were densely filled with red‐stained lipids, while both L‐IXN and H‐IXN groups exhibited a remarkable reduction in lipid accumulation. Notably, the liver tissues of the H‐IXN group displayed fewer red‐stained lipids, suggesting a dose‐dependent effect (Figure 2b,d). Consistent with H&E and ORO staining, hepatic TG and TC levels mirrored these improvements (Figure 2e,f), demonstrating IXN's potential in mitigating Triton‐induced hepatic lipid abnormalities.

### 3.3 PPARα and AKT2 serve as potential therapeutic targets of IXN

#### 3.3.1 Target prediction and PPI network construction

**FIGURE 3:**
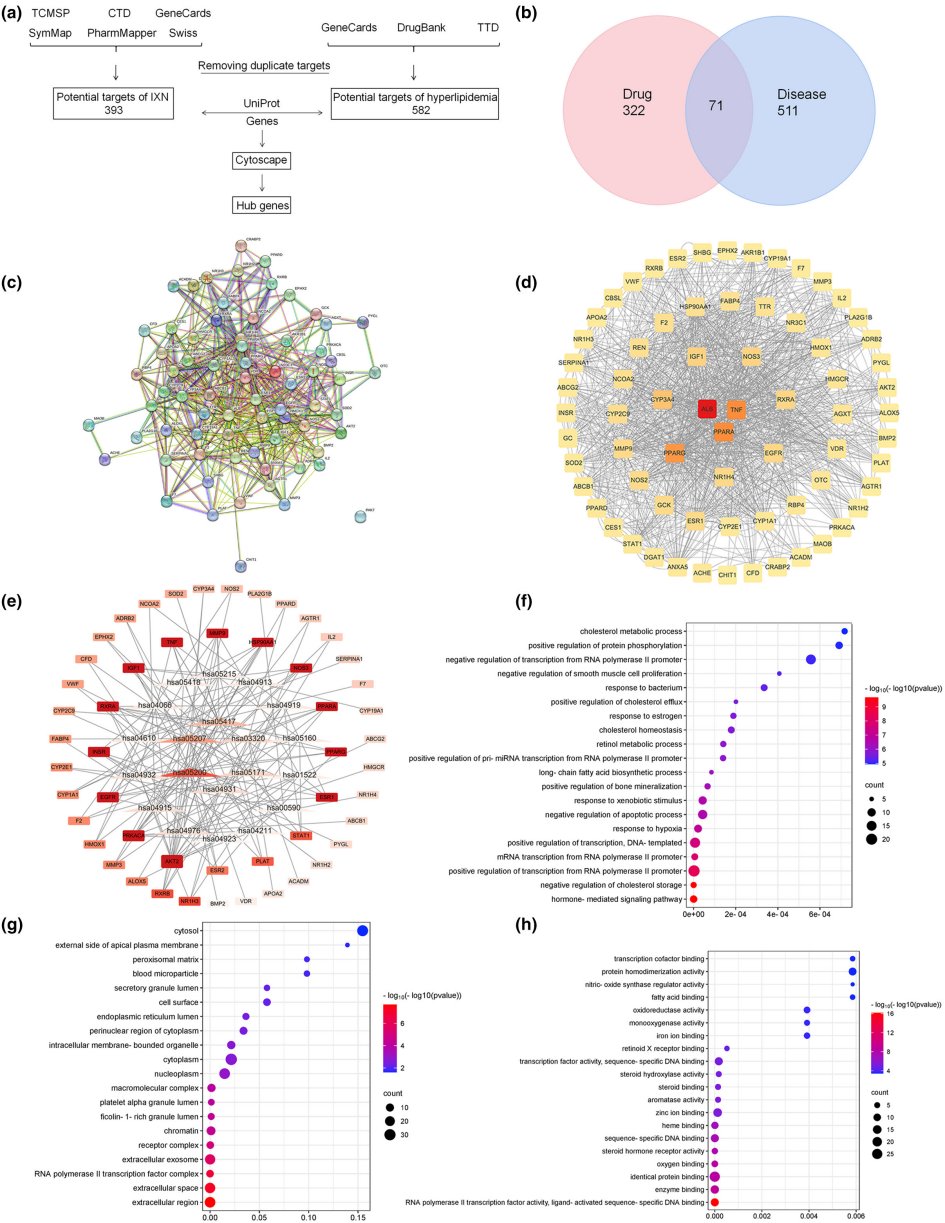
Screening of targets of IXN against hyperlipidemia. (a) Flowchart for screening core targets. (b) Intersected target genes of IXN and hyperlipidemia. (c) Protein–protein interaction (PPI) network of intersected target genes. (d) PPI network of key target genes based on betweenness centrality. (e) The top 20 items of KEGG pathway and core gene topology analysis. (f) The top 20 items of biological process. (g) The top 20 items of cellular composition. (h) The top 20 items of molecular function. IXN, isoxanthohumol; KEGG, Kyoto Encyclopedia of Genes and Genomes.

A systematic approach was employed for target prediction and PPI network construction, as outlined in the flowchart depicted in Figure 3a. Targets of IXN were predicted from various databases, yielding 393 potential targets. Concurrently, hyperlipidemia‐related targets were collected from different databases, resulting in 582 potential targets. The intersection of IXN and hyperlipidemia targets revealed 71 shared targets (Figure 3b). These core targets were then subjected to PPI network analysis through the string database, elucidating the interrelation among differential proteins (Figure 3c,d). Colors represented BC values from high (red) to low (yellow). Notably, the top 10 targets included albumin (ALB), tumor necrosis factor (TNF), peroxisome proliferator‐activated receptor alpha (PPARA), peroxisome proliferator‐activated receptor gamma (PPARG), cytochrome P450 3A4 (CYP3A4), insulin‐like growth factor 1 (IGF1), nitric oxide synthase 3 (NOS3), retinoid X receptor alpha (RXRA), epidermal growth factor receptor (EGFR), and farnesoid X receptor (NR1H4).

#### 3.3.2 Functional enrichment analysis

KEGG enrichment analysis indicated the involvement of candidate genes in lipid metabolism‐related pathways such as PPAR signaling pathway (hsa03320), lipid and atherosclerosis (hsa05417), nonalcoholic fatty liver disease (hsa04932), regulation of lipolysis in adipocytes (hsa04923), and arachidonic acid metabolism (hsa00590) (Table S2). Targets with the deepest (red) color were considered as the hub targets in the network (Figure 3e). Threonine‐protein kinase (AKT2) emerged as the top core gene among key genes like cAMP‐dependent protein kinase catalytic subunit alpha (PRKACA), EGFR, insulin receptor (INSR), RXRA, IGF1, TNF, matrix metalloproteinase‐9 (MMP9), heat shock protein HSP 90‐alpha (HSP90AA1), NOS3, and PPARα.

Furthermore, we performed GO enrichment in each cluster and selected the top 20 GO terms with *p*‐value to visualize the results. The depth of color represented enrichment *p*‐value (−log 10). The size of the bubble represented the number of enriched genes. The most significantly enriched BP terms were associated with negative regulation of cholesterol storage, response to hypoxia, response to xenobiotic stimulus, negative regulation of apoptotic process, long‐chain fatty acid biosynthetic process, cholesterol homeostasis, positive regulation of cholesterol efflux, positive regulation of protein phosphorylation, and cholesterol metabolic process. Regarding CC, the targets were mainly enriched in extracellular region, extracellular space, RNA polymerase II transcription factor complex, extracellular exosome, receptor complex, chromatin, ficolin‐1‐rich granule lumen, platelet alpha granule lumen, macromolecular complex, and nucleoplasm. At the level of MF aspect, RNA polymerase II transcription factor activity, ligand‐activated sequence‐specific DNA binding, enzyme binding, oxygen binding, identical protein binding, and steroid hormone receptor activity were significantly enriched (Figure 3f–h).

#### 3.3.3 Molecular docking analysis

A recent study demonstrated that the AMPK/PPARα signaling pathway was implicated in hepatic steatosis (Arulkumar et al., 2022). Existing pharmaceutical agents known to ameliorate disturbances in lipid metabolism have been empirically shown to predominantly exert their regulatory effects by activating PI3K/AKT signaling pathway (Zhang et al., 2022; Zhou et al., 2021). In conjunction with the aforementioned the network of target signaling pathways and enrichment analysis of KEGG pathways, we posited that PPARα and AKT2 represented potential targets influenced by IXN.

**FIGURE 4:**
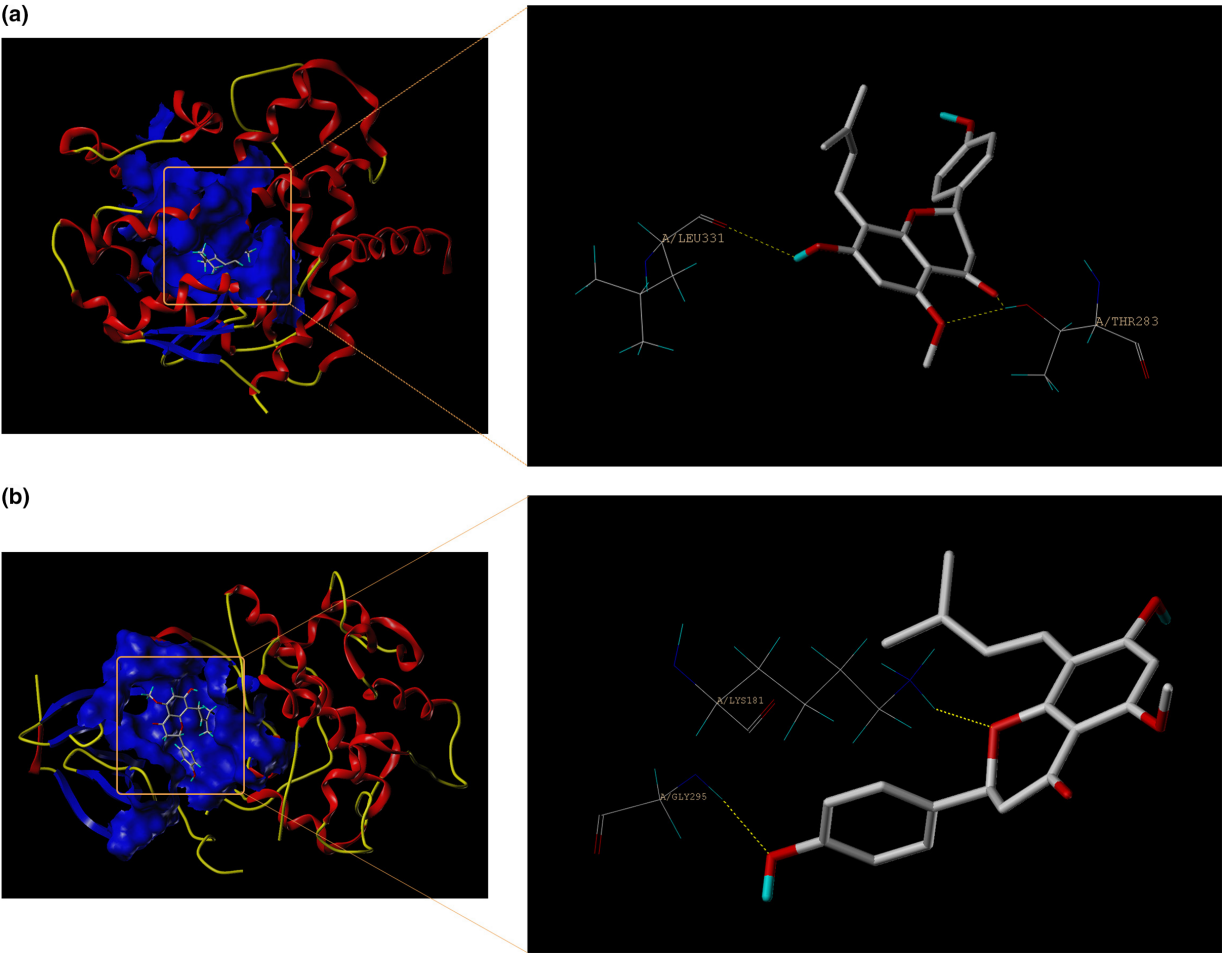
Docking mode between IXN and associated key targets. (a) The docking conformation between IXN and PPARα. (b) The docking conformation between IXN and AKT2. IXN, isoxanthohumol.

The crystal structures of PPARα (PDB ID: 2rew) and AKT2 (PDB ID: 1gzn) were downloaded from the RCSB Protein Data Bank (PDB) (http://www.rcsb.org). The modification of these crystal structures, including removing ligand and water, as well as adding hydrogen and the missing amino acid residues, was performed using the Sybyl 2.0 software. The 3D structure‐data file of IXN was retrieved from PubChem database. Then, the Sybyl 2.0 software was performed for all docking simulations, and Surflex Dock docking mode was performed to obtain Surflex Dock score indicating binding affinity. The resulting docking scores for PPARα and AKT2 were 5.8180 and 6.1157, respectively, suggesting strong and substantiated interactions between IXN and these proteins (Figure 4 and Table S3).

### 3.4 Regulatory effect of IXN on AMPK/PPARα and PI3K/AKT signaling pathways

The prediction outcomes from our network pharmacology analysis and molecular docking suggested that PPARα and AKT were pivotal targets in IXN against hyperlipidemia. We have further conducted experiments to observe the effects of AKT agonist (SC79) on hyperlipidemia. As shown, SC79 (0.04 mg/g, i.p.) improved serum parameters and hepatic lipid accumulation to some extent (Figure S2) (Jo et al., 2012). Collectively, our results illustrated the impact of PPARα agonist (Fenofibrate) and AKT agonist (SC79) on hyperlipidemia.

**FIGURE 5:**
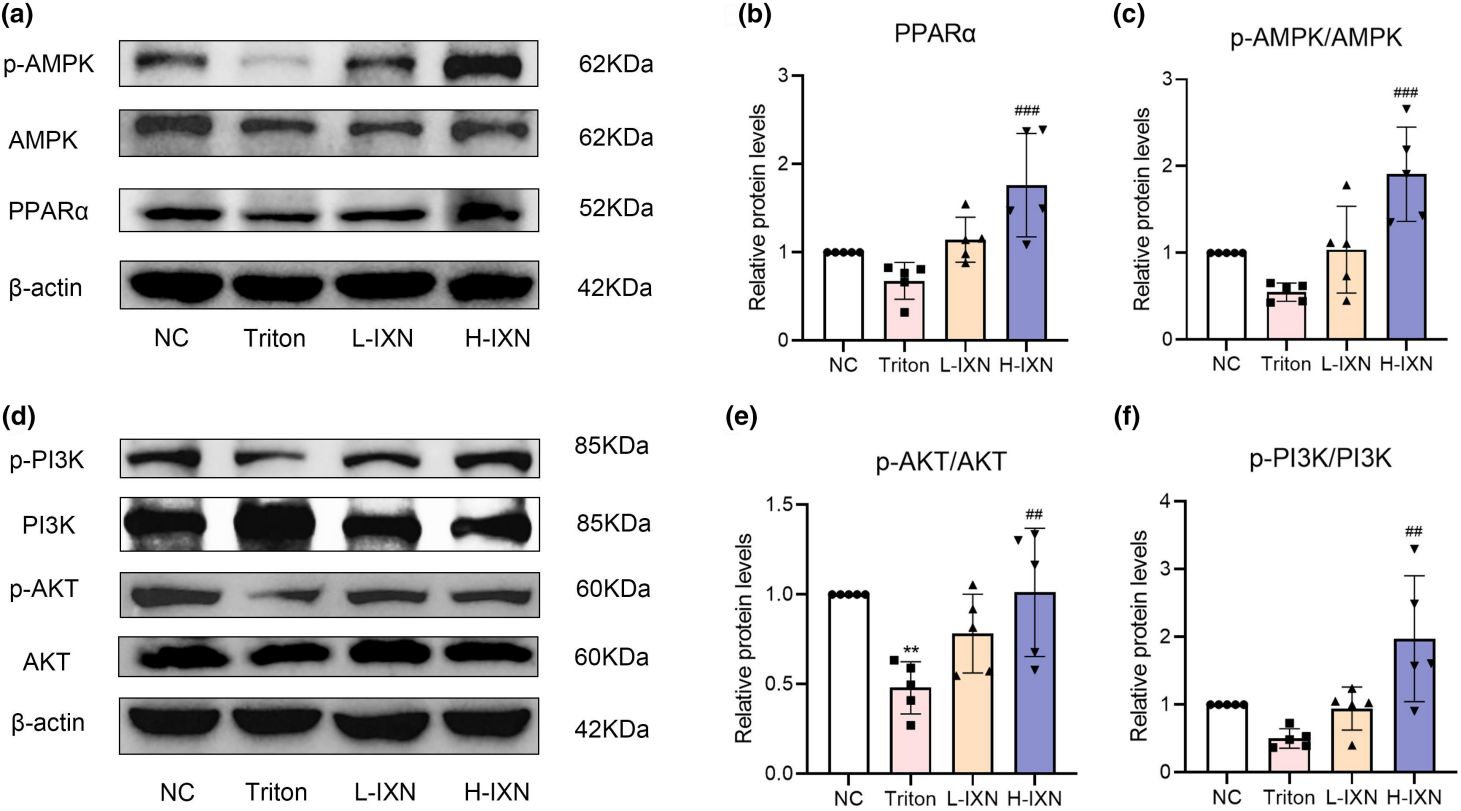
Effect of IXN on AMPK, p‐AMPK, PPARα, PI3K, p‐PI3K, AKT, and p‐AKT protein expression levels in hyperlipidemic mice liver tissue. (a) Representative bands of AMPK, p‐AMPK, and PPARα (inner reference: β‐Actin). (b) Relative protein levels of PPARα. (c) Relative protein levels of p‐AMPK/AMPK. (d) Representative bands of PI3K, p‐PI3K, AKT, and p‐AKT (inner reference: β‐Actin). (e) Relative protein levels of p‐AKT/ AKT. (f) Relative protein levels of p‐PI3K/PI3K. **p < .01 versus NC group. ^##^ *p* < .01, ^###^ *p* < .001 versus triton group. IXN, isoxanthohumol.

Next, we experimentally verified differential expression of PPARα and AKT through WB. Concurrently, the upstream signaling proteins (AMPK and PI3K) were also evaluated by WB. IXN groups exhibited significantly increased hepatic p‐AMPK, PPARα, p‐PI3K, and p‐AKT protein expression levels compared with the Triton group (Figure 5). Our findings strongly suggested that IXN upregulated the hepatic AMPK/PPARα and PI3K/AKT signaling pathways in hyperlipidemic mice, supporting the molecular basis for its therapeutic effects.

## 4 DISCUSSION

Hyperlipidemia stands out as a modifiable risk factor with significant implications for cardiovascular and cerebrovascular diseases. Controlling blood lipid concentrations and enhancing liver lipid metabolism are paramount in preventing severe health conditions (Deprince et al., 2020; Miller et al., 2011). The conventional lipid regulators, statins, and fibrates, though widely used, carry the risk of drug‐induced liver and kidney damage (Liu et al., 2019; Okopień et al., 2018). As such, there is a critical need to explore novel therapeutics with potential therapeutic value and minimal side effects. Our study establishes that IXN significantly improves blood lipid levels, attenuates hepatic steatosis, enhances antioxidant enzyme activity, and reduces MDA levels. These beneficial effects are possibly attributed to the upregulation of AMPK/PPARα and PI3K/AKT signaling pathways.

Moderate alcohol intake is related to lower rates of cardiovascular events and improved metabolic health (Marcos et al., 2021). Dietary interventions using natural compounds might be a candidate for an adjuvant therapy for hyperlipidemiaI. IXN, derived from the brewing process of hops, represents a common flavonoid in hopped beers (Stevens et al., 1999), yet its effects on hyperlipidemia remain underexplored. Our study comprehensively investigates the therapeutic potential of IXN, exploring serum biochemical changes, antioxidant enzyme activity, and histopathological alterations.

The Triton WR‐1339 model was extensively utilized as the rapid and convenient system for screening natural or chemical lipid‐lowering drugs and has been successfully employed to investigate serum and liver lipid metabolism in various animal models (Xie et al., 2007; Xu et al., 2019; Zhu et al., 2000). In our study, the Triton‐induced hyperlipidemia model validated by elevated serum TC and TG levels, along with histological evidence of hepatic vacuolization and lipid droplets, underscored the perturbed lipid metabolism. Notably, IXN treatment effectively mitigated these Triton‐induced adverse effects. Furthermore, our findings align with the correlation between hyperlipidemia and oxidative stress (Ben Hamad Bouhamed et al., 2019; Yang et al., 2008), demonstrating that IXN consumption reduced MDA levels and enhanced SOD activities, mitigating oxidative damage induced by Triton.

In‐depth exploration through network pharmacology and molecular docking elucidated potential mechanisms underlying IXN's anti‐hyperlipidemic effects. The analysis of biological processes, targets, and signaling pathways related to hyperlipidemia highlighted lipid metabolism‐related processes as significantly enriched. In particular, PPARα was the hub target in protein–protein interaction network, while AKT acted as a crucial bridging protein in KEGG enrichment analysis.

PPARα, identified as a primary transcriptional regulator, modulates genes integral to fatty acid oxidation and lipid metabolism. Previous study has emphasized that increasing AMPK phosphorylation and upregulating PPARα can mitigate impaired lipid metabolism (Bordoloi et al., 2019). Furthermore, AKT, recognized as a critical and versatile protein kinase, orchestrates nutrient uptake and metabolism intrinsically within cells through various downstream targets (Manning & Toker, 2017). AKT activation inhibits lipogenesis and excessive lipid accumulation in the liver (Cao et al., 2016). Also, PI3K/AKT pathway is intricately associated with cholesterol metabolism (Zhou et al., 2014). Studies have reported the amelioration of dyslipidemia and oxidative stress in a model of metabolic syndrome through the upregulation of PI3K/AKT (Ajala‐Lawal et al., 2020). Additionally, the activated AMPK significantly influences the PI3K/AKT signaling pathway (Zhou et al., 2021), reinforcing the interconnected nature of these pathways. Interestingly, according to our results, PPARα agonist (Fenofibrate) exerted a more potent effect compared with AKT agonist (SC79) (Figures 1 and 2, and Figure S2). This may imply that AMPK/ PPARα pathway played a more dominant role than PI3K/AKT signaling pathway in hyperlipidemic mice.

In alignment with these findings, our molecular docking results underscored the robust binding capacity of IXN with both PPARα and AKT. To further delve into the effects of IXN on AMPK/PPARα and PI3K/AKT signaling pathways in hyperlipidemic mice, we conducted WB comparing protein expression levels between the IXN and Triton groups. The results unequivocally demonstrated that IXN significantly increased the expression levels of PPARα, p‐AMPK, p‐AKT/AKT, and p‐PI3K/PI3K, effectively counteracting the adverse effects induced by Triton. This substantiates the therapeutic potential of IXN in ameliorating hyperlipidemia through intricate modulation of key signaling pathways associated with lipid metabolism. In particular, compared to a single target, a target combination may be a better strategy for treating hyperlipidemia.

Although our results demonstrate that IXN has anti‐hyperlipidemia effects by activating AMPK/PPARα and PI3K/AKT pathways to mitigate lipid accumulation and oxidative stress, it is imperative to recognize certain limitations. First of all, the pharmacological effects of IXN may involve not only predicted targets but also hyperlipidemic targets in the database. Some bias may exist due to the different computer algorithms used in each database. Second, an in‐depth exploration of the downstream signaling pathways associated with AMPK/PPARα and PI3K/AKT is necessary to fully understand the molecular mechanisms underlying IXN's therapeutic effects. Lastly, our animal model was an acute model that did not adapt to long‐term treatment. Therefore, we could not clearly assess the chronic side effects of IXN. To enhance the generalizability of the study, the evaluation of IXN's therapeutic efficacy should be extended to encompass multiple animal models of hyperlipidemia. Addressing these limitations will contribute to a more robust understanding of IXN's potential and aid in refining its application as a therapeutic intervention for hyperlipidemia.

## 5 CONCLUSION

This study reveals IXN's potent anti‐hyperlipidemic effects by dissecting its impact on AMPK/PPARα and PI3K/AKT pathways. The integration of experimental and computational methods advances understanding of targeted drug development in hyperlipidemia. This comprehensive approach bridges traditional wisdom with modern science, offering pivotal insights into novel treatment strategies for intractable diseases.

## AUTHOR CONTRIBUTIONS

**Yu Gao:** Conceptualization (lead); data curation (lead); formal analysis (lead); methodology (lead); software (lead); validation (lead); visualization (lead); writing – original draft (lead). **Qilong Zhou:** Conceptualization (equal); data curation (lead); methodology (lead); project administration (lead). **Huiqing Wang:** Funding acquisition (lead); investigation (lead); resources (supporting). **Guang Xin:** Project administration (lead); supervision (lead); writing – original draft (lead). **Tao Wang:** Writing – original draft (lead). **Kun Zhang:** Resources (lead). **Xiuxian Yu:** Resources (lead). **Ao Wen:** Resources (lead). **Qiuling Wu:** Writing – original draft (supporting). **Xiaojuan Li:** Software (supporting). **Yijiang Liu:** Software (supporting). **Wen Huang:** Funding acquisition (lead); investigation (lead); supervision (lead).

## FUNDING INFORMATION

This research was supported by the Innovative Chinese Medicine and Health Products Research Academician Workstation of Academicians Boli Zhang and Beiwei Zhu of the West China Hospital, Sichuan University (No. HXYS19001, HXYS19002), the 135 Project for Disciplines of Excellence, West China Hospital, Sichuan University (No. ZYXY21002), and the Innovative Chinese Medicine Preclinical Research Fund of “Liqing No.2,” West China Hospital, Sichuan University (No. 161200012).

## CONFLICT OF INTEREST STATEMENT

The authors declare no conflicts of interest.

## Supporting information


Data S1.

## Data Availability

The data that support the findings of this study are available from the corresponding author upon reasonable request.
