# Supplementary material for: Isoxanthohumol improves hepatic lipid metabolism via regulating the AMPK/PPARα and PI3K/AKT signaling pathways in hyperlipidemic mice
Source: Food Sci Nutr. 2024 Sep 10;12(11):8846–57. doi: 10.1002/fsn3.4449 (PMC11606862; doi:10.1002/fsn3.4449)
Supplement: Supplementary file 1 — Data S1. [file FSN3-12-8846-s001.docx]

# SUPPLEMENTARY MATERIALS

**Table S1** Non-Alcoholic Steatohepatitis Clinical Research Network Scoring System

| Steatosis grade  (0-3) + | Lobular inflammation  (0-3) + | Hepatocyte ballooning  (0-2) |
| --- | --- | --- |
| 0: <5% | 0: None | 0: None |
| 1:5-33% | 1:<2 foci/20x field | 1: Mild, few |
| 2: 34-66% | 2: 2-4 foci/20x field | 2: Moderate - Many |
| 3: >66% | 3: >4 foci/20x field |  |
| nonalcoholic fatty liver disease activity score (NAS): 0-8 |  |  |

**Table S2** The KEGG pathway corresponding to the hsa code

| ID code | Pathway |
| --- | --- |
| hsa05207 | Chemical carcinogenesis - receptor activation |
| hsa05200 | Pathways in cancer |
| hsa03320 | PPAR signaling pathway |
| hsa05417 | Lipid and atherosclerosis |
| hsa04915 | Estrogen signaling pathway |
| hsa04931 | Insulin resistance |
| hsa04913 | Ovarian steroidogenesis |
| hsa04976 | Bile secretion |
| hsa05215 | Prostate cancer |
| hsa01522 | Endocrine resistance |
| hsa04932 | Non-alcoholic fatty liver disease |
| hsa04066 | HIF-1 signaling pathway |
| hsa04919 | Thyroid hormone signaling pathway |
| hsa05171 | Coronavirus disease - COVID-19 |
| hsa04610 | Complement and coagulation cascades |
| hsa04211 | Longevity regulating pathway |
| hsa05418 | Fluid shear stress and atherosclerosis |
| hsa04923 | Regulation of lipolysis in adipocytes |
| hsa00590 | Arachidonic acid metabolism |
| hsa05160 | Hepatitis C |

**Table S3** Molecule docking score

| Gene | Total_Score | Crash | Polar |
| --- | --- | --- | --- |
| PPARA | 5.8180 | -2.9974 | 1.6288 |
| AKT2 | 6.1157 | -1.2342 | 1.1301 |


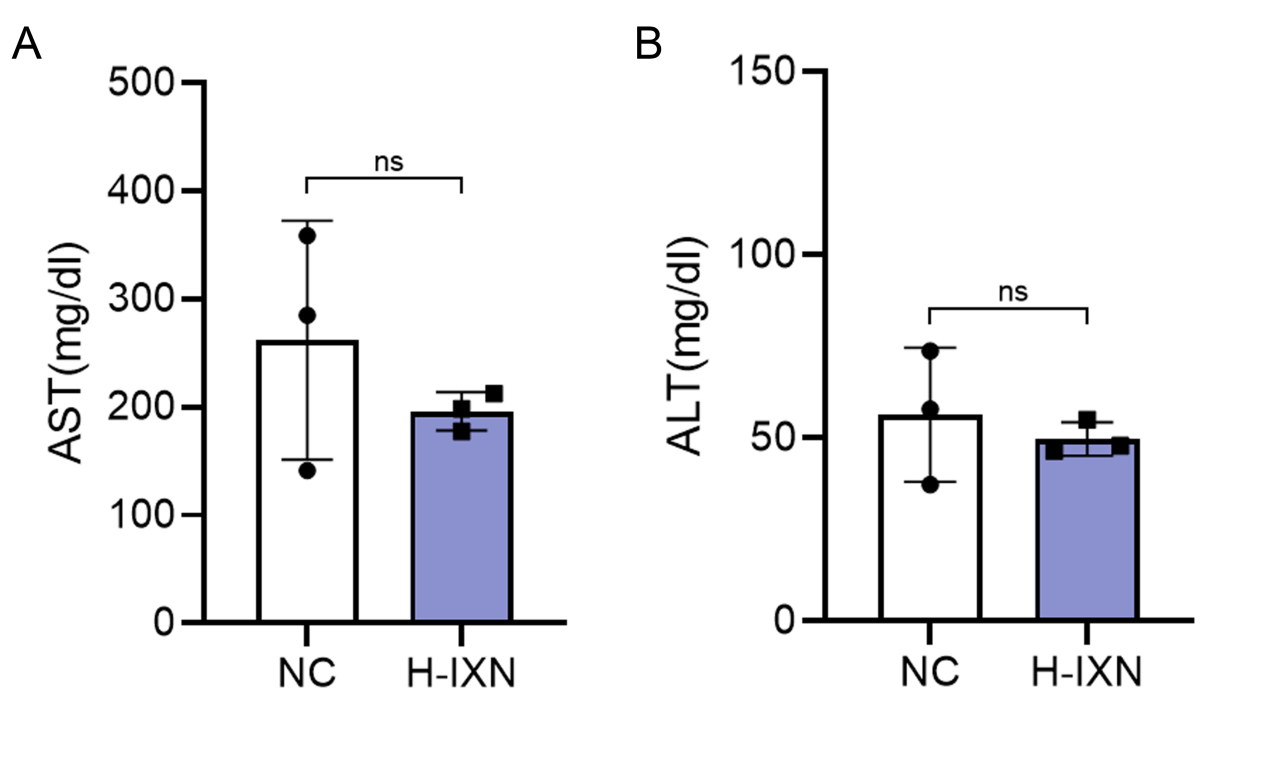


**Figure S1** Effects of IXN alone administration on mice serum AST and ALT. (**A**) Serum AST. (**B**) Serum ALT. Data are shown as mean ± SD for n = 3. NS: denotes no significant difference.


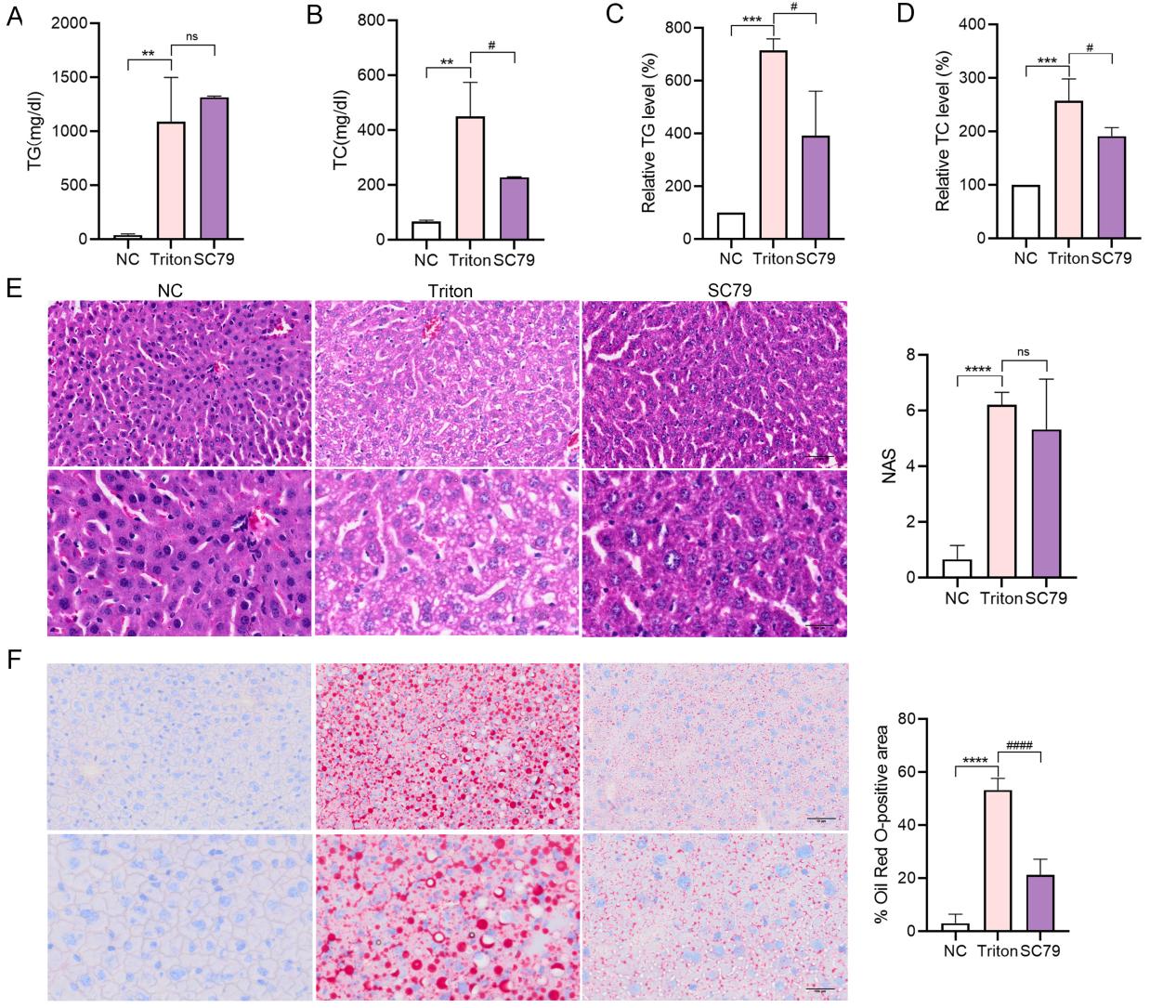


**Figure S2** Effects of SC79 on serum lipids and histological changes in liver tissues. (**A**) Serum TG. (**B**) Serum TC. (**C**) Hepatic triglyceride content. (**D**) Hepatic total cholesterol. (**E**) Representative photomicrographs of liver histology (H&E) and NAS from each group. Scale bar, 50 μm and 100 μm (insets). (**F**) Oil Red O staining of lipid droplets liver and quantification of relative staining area. Scale bar, 50 μm and 100 μm (insets). All data represent mean ± SEM. ***P* < 0.01, ****P* < 0.001, *****P* < 0.0001 vs. NC group. ^#^*P* < 0.05, ^####^*P* < 0.0001 vs. Triton group. NS: denotes no significant difference.
